# Supplementary figures and images for: Advanced Glycation End-Product-Modified Heat Shock Protein 90 May Be Associated with Urinary Stones
Source: Diseases. 2025 Jan 2;13(1):7. doi: 10.3390/diseases13010007 (PMC11764404; doi:10.3390/diseases13010007)

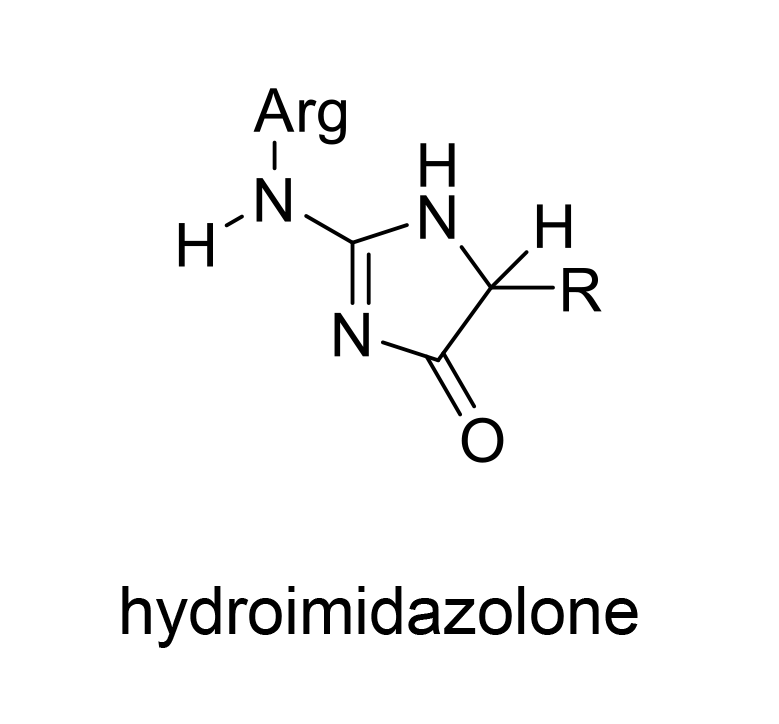

Supplement: Supplementary file 1 [file diseases-13-00007-s001.zip › Diseases_Suppl_Figure_S1_.tif]

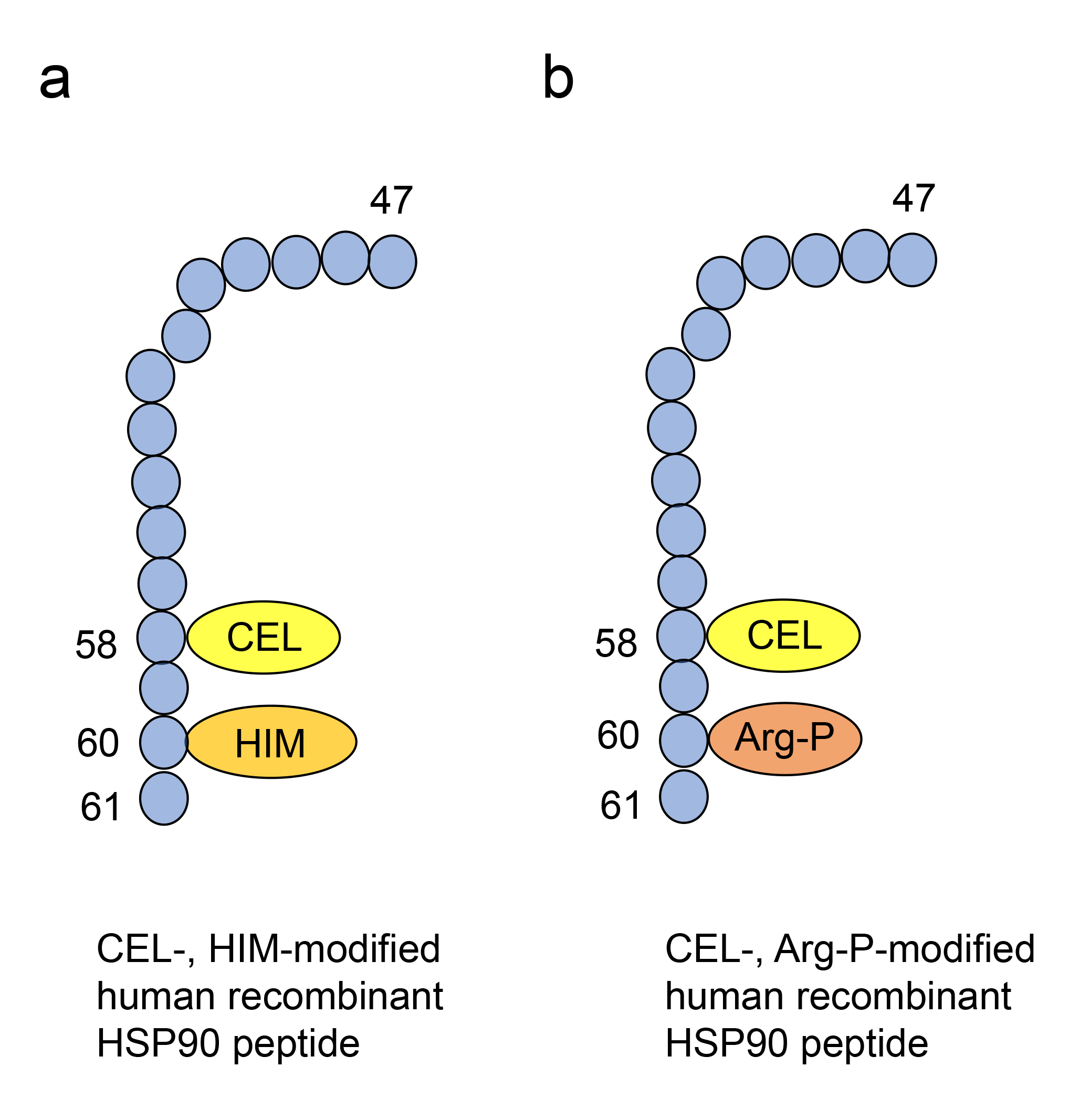

Supplement: Supplementary file 1 [file diseases-13-00007-s001.zip › Diseases_Suppl_Figure_S2_.tif]

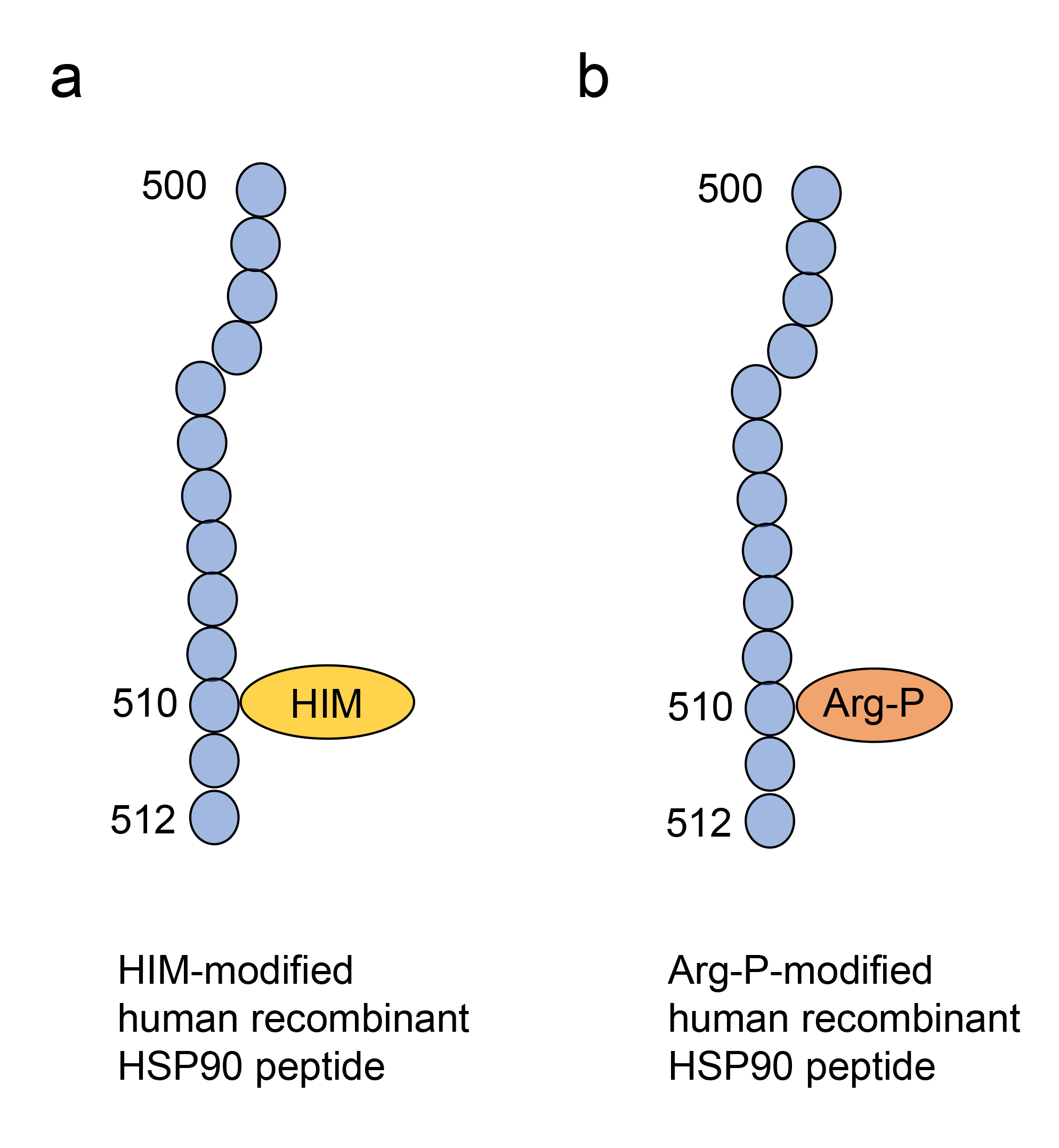

Supplement: Supplementary file 1 [file diseases-13-00007-s001.zip › Diseases_Suppl_Figure_S3_.tif]

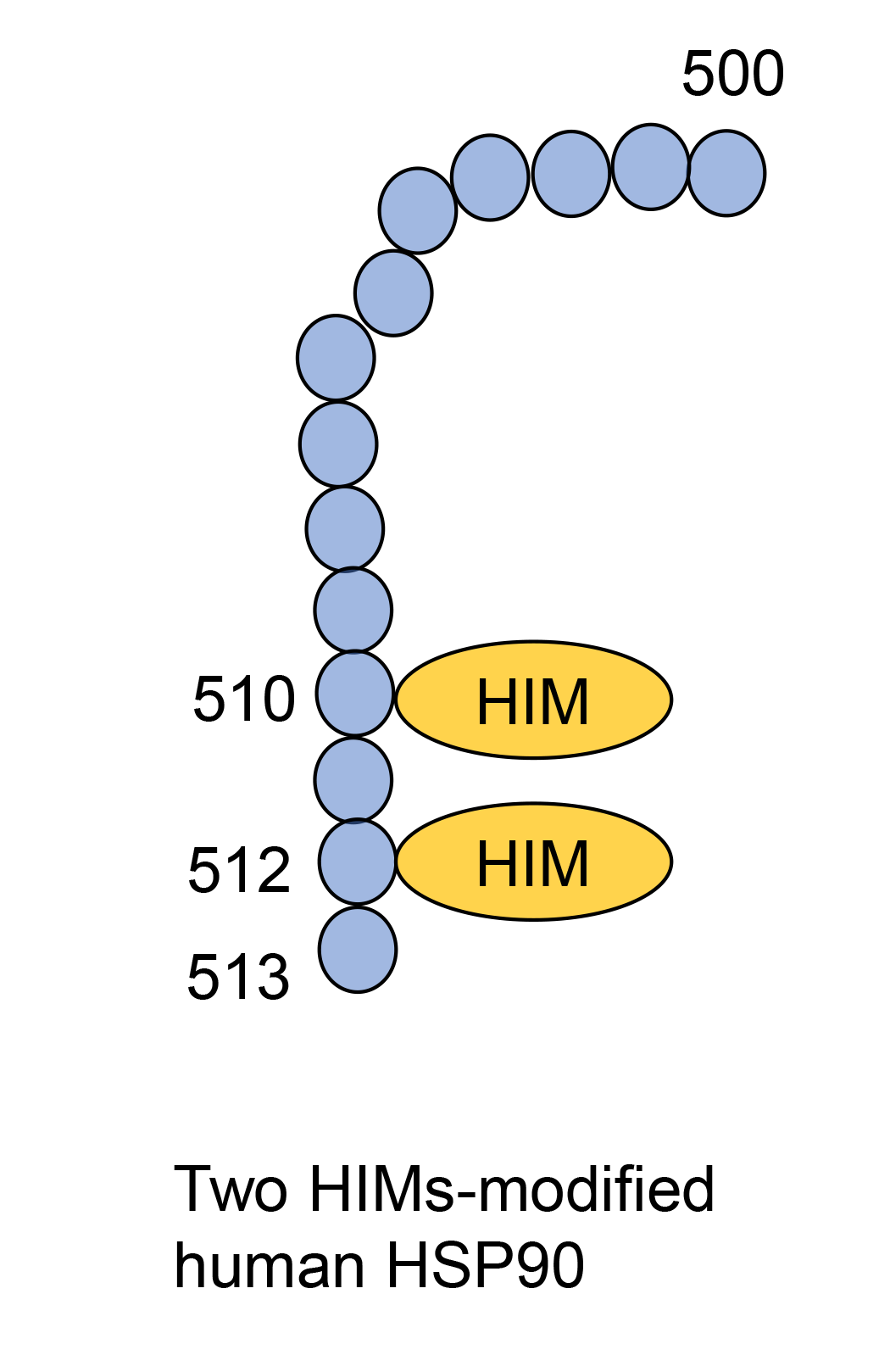

Supplement: Supplementary file 1 [file diseases-13-00007-s001.zip › Diseases_Suppl_Figure_S4_.tif]
